# Incidence of First Stroke in Pregnant and Nonpregnant Women of Childbearing Age: A Population‐Based Cohort Study From England

**DOI:** 10.1161/JAHA.116.004601

**Published:** 2017-04-21

**Authors:** Lu Ban, Nikola Sprigg, Alyshah Abdul Sultan, Catherine Nelson‐Piercy, Philip M. Bath, Jonas F. Ludvigsson, Olof Stephansson, Laila J. Tata

**Affiliations:** ^1^ Division of Epidemiology & Public Health University of Nottingham Nottingham United Kingdom; ^2^ Division of Rheumatology, Orthopaedics and Dermatology Centre of Evidence Based Dermatology University of Nottingham Nottingham United Kingdom; ^3^ Stroke Division of Neuroscience University of Nottingham Nottingham United Kingdom; ^4^ Arthritis Research UK Primary Care Centre Research Institute for Primary Care & Health Sciences Keele University Keele United Kingdom; ^5^ Women's Health Academic Centre Guy's and St. Thomas' NHS Foundation Trust London United Kingdom; ^6^ Department of Medical Epidemiology and Biostatistics Karolinska Institutet Stockholm Sweden; ^7^ Department of Medicine Karolinska Institutet Stockholm Sweden; ^8^ Department of Women's and Children's Health Karolinska Institutet Stockholm Sweden; ^9^ Department of Paediatrics Örebro University Hospital Örebro Sweden

**Keywords:** epidemiology, pregnancy and postpartum, stroke, women, Epidemiology, Women, Pregnancy, Cerebrovascular Disease/Stroke

## Abstract

**Background:**

Pregnant women may have an increased risk of stroke compared with nonpregnant women of similar age, but the magnitude and the timing of such risk are unclear. We examined the risk of a first stroke event in women of childbearing age and compared the risk during pregnancy and in the early postpartum period with the background risk outside these periods.

**Methods and Results:**

We conducted an open cohort study of 2 046 048 women aged 15 to 49 years between April 1, 1997, and March 31, 2014, using linked primary (Clinical Practice Research Datalink) and secondary (Hospital Episode Statistics) care records in England. Risk of first stroke was assessed by calculating the incidence rate of stroke in antepartum, peripartum (2 days before until 1 day after delivery), and early (first 6 weeks) and late (second 6 weeks) postpartum periods compared with nonpregnant time using a Poisson regression model with adjustment for maternal age, socioeconomic group, and calendar time. A total of 2511 women had a first stroke. The incidence rate of stroke was 25.0 per 100 000 person‐years (95% CI 24.0–26.0) in nonpregnant time. The rate was lower antepartum (10.7 per 100 000 person‐years, 95% CI 7.6–15.1) but 9‐fold higher peripartum (161.1 per 100 000 person‐years, 95% CI 80.6–322.1) and 3‐fold higher early postpartum (47.1 per 100 000 person‐years, 95% CI 31.3–70.9). Rates of ischemic and hemorrhagic stroke both increased peripartum and early postpartum.

**Conclusions:**

Although the absolute risk of first stroke is low in women of childbearing age, healthcare professionals should be aware of a considerable increase in relative risk during the peripartum and early postpartum periods.

## Introduction

Stroke is a leading cause of death in high‐income countries including the United Kingdom.^1^ Although the risk of stroke among women aged <40 years is relatively low compared with older women, pregnancy can substantially increase this risk.^1^ Estimates of the absolute risk of stroke in and around pregnancy are thus crucial for planning healthcare resources and decision making. There is, however, large variation in the reported incidence of stroke in pregnant women^2^; reports vary with 1.5 per 100 000 deliveries in the United Kingdom,^3^ 21.5 per 100 000 deliveries in Taiwan,^4^ and 34.2 per 100 000 deliveries in the United States.^5^ Previous large population‐based studies did not provide a complete picture of stroke incidence by either missing women in the early antepartum^6^, ^7^, ^8^ or postpartum periods^3^ or by not distinguishing between risks in antepartum and postpartum periods.^9^, ^10^

The recent World Stroke Day campaigns in 2014–2016 highlighted the impact of stroke on women and emphasized the importance of preventing stroke in women of all ages in the United Kingdom.^11^ Nevertheless, to our knowledge, there has been no population‐based study in the United Kingdom to examine the risk of stroke in pregnancy and postpartum compared with nonpregnant periods in women of childbearing age, which is important for planning preventative strategies. The primary care Clinical Practice Research Datalink (CPRD) and Hospital Episode Statistics (HES) linked data have given us a unique opportunity to quantify the population‐level absolute risk of stroke in specific antepartum and postpartum periods by utilizing data on >2 million women. The aim of this study was to quantify the incidence rates of ischemic stroke, intracerebral hemorrhage, and subarachnoid hemorrhage in women of childbearing age and to compare the rates antepartum and in the early postpartum period with the background rates outside these periods.

## Methods

### Database and Study Population

The CPRD is a routinely collected electronic primary care database of anonymized medical records from >650 general practitioners across the United Kingdom. Approximately 7% of the UK population is included, and the data are broadly representative of the UK general population in terms of age and sex.^12^ General practitioners are gatekeepers of the National Health Service (NHS) in the United Kingdom and are the primary point of contact for nonemergency health care. Around 98% of the UK population is registered with a general practitioner. CPRD contains demographic, medical, prescription, and lifestyle‐related information recorded using the Read code system^13^ and has been extensively validated for a wide range of diagnoses for epidemiological research.^14^ For this study, we used a subset of English practices within CPRD that have been linked to the HES, containing details of all hospital admissions to NHS hospitals in England. About 58% of CPRD practices are linked to HES; the linkage is done by a trusted third party using NHS number, date of birth, and sex. Because HES covers only English hospitals, practices from Northern Island, Wales, and Scotland are excluded for the linkage. The CPRD‐HES linked data have been compared with Office for National Statistics (ONS) data showing similar age and sex distributions.^15^ Along with basic demographic information, HES contains information on discharge diagnoses (including 1 primary diagnosis and up to 19 secondary/subsidiary diagnoses) and procedures that are coded using the *International Classification of Diseases, 10th revision* (ICD‐10), and Operation and Procedure Coding Supplement version 4, respectively. Particularly, Maternity HES contains information on all births across England and has been validated against birth registration data and NHS Numbers for Babies data.^16^

We used an open cohort study design including all women aged 15 to 49 years from the CPRD‐HES between April 1, 1997, and March 31, 2014. The follow‐up start date for each woman was defined as the start of the CPRD‐HES link (April 1, 1997), the date of a woman becoming 15 years old, the date of a woman's registration with a general practice, or the up‐to‐standard date of that practice, whichever came latest; the follow‐up end date was defined as the last date for CPRD‐HES link (March 31, 2014), the date before a woman became 50 years old, the date of a woman transferring out of the practice, the date of a woman's death, or the last date of data collection, whichever came earliest. Women with a prior history of stroke before the study start date were excluded from this cohort study.

### Defining Exposure Time

For each woman, we extracted information on pregnancy outcome (live birth or stillbirth, date of delivery, and gestation) from Maternity HES. In the United Kingdom, stillbirth is defined as a baby deceased after 24 completed weeks of pregnancy. Women's follow‐up time between ages 15 and 49 years was divided into time associated with pregnancy (defined from the date of conception until 12 weeks postpartum) and nonpregnant time (all other available follow‐up time, which included all time for women who were never pregnant during the study period, as previously defined^17^).

The time associated with pregnancy was divided into antepartum (from the date of conception until 3 days before the date of delivery), peripartum (2 days before until 1 day after delivery), and postpartum (from 2 days after delivery until the end of the 12th week postpartum). The period of 2 days before until 1 day after delivery was defined as peripartum based on a previous Swedish study that found the risk of stroke was high during this period.^6^ The antepartum period was further subdivided into trimesters, and the postpartum period was further subdivided into individual weeks and into early (first 6 weeks) and late (second 6 weeks) postpartum.

### Outcome

The outcome of this study was first incident stroke during the study period. Stroke diagnosis was identified using ICD‐10 codes (I60–I64, O22.5, and O87.3) from hospital records or Read codes from primary care records. We also used ONS death records linked to CPRD‐HES to identify stroke that had resulted in death and may have been diagnosed only postmortem. Cause of death for stroke was recorded in the ONS data using first ICD‐9 (430–434, 436, and 437) and then ICD‐10 (I60–I64, O22.5, and O87.3) codes during the study period. We included only deaths that had stroke as the primary cause of death. Because women with a prior history of stroke may have a risk of recurrent stroke and may have been on secondary prevention to reduce this risk, we excluded women if they had a history of stroke before the start of the study period. Women with an incident stroke during the study period were followed until time of the first event for a similar reason, namely, women's clinical follow‐up and personal health could be subsequently modified by this event, which could in turn modify the occurrence of a subsequent stroke event. We also excluded women if the first stroke event was recorded in CPRD primary care data within the first month of a woman's registration with her current general practice but without proximal hospital admission within 30 days or if the first stroke event recorded in HES was not the primary or the first 2 secondary diagnoses, as these might be a recording of medical history.

Stroke events were classified as ischemic stroke (IS), intracerebral hemorrhage (ICH), and subarachnoid hemorrhage (SAH). The type of stroke was identified from the ICD codes in the HES record if a woman's first stroke diagnosis was in HES or if she was hospitalized for stroke within 30 days after a first stroke diagnosis in primary care data. Read codes in the CPRD record were used to identify stroke type if the woman's first stroke diagnosis was in CPRD and she had no hospitalization within 30 days. Although it is possible to have more than one type of stroke in close succession (eg, ICH after acute IS or infarction after SAH),^18^ our aim was to identify first incident stroke. Consequently, for women with different types of stroke within the same hospitalization, only the earliest diagnosis was used to identify the stroke type. In addition, some women whose stroke type was unspecified were classified as having IS if they had a prescription for an antiplatelet or an anticoagulant drug from primary care within 2 months after the diagnosis. Finally, women with multiple types of stroke but no clear indication of the diagnosis order were classified as having stroke type unspecified.

### Statistical Analysis

We calculated the absolute rates of stroke per 100 000 person‐years with 95% CIs for all time periods and for different types of stroke. Poisson regression was used to estimate incidence rate ratios (IRRs) of stroke in antepartum and postpartum periods compared with the nonpregnant time period with adjustment for maternal age (15–24, 25–34, and 35–49 years), socioeconomic deprivation (defined as quintiles of 2010 English Index of Multiple Deprivation), and calendar time (1997–2002, 2003–2008, 2009–2014). Age and calendar time were treated as time‐varying covariates created using Lexis expansion.^19^ We also presented stratified analysis by each of these covariates with mutual adjustment for the rest of the covariates. The English Index of Multiple Deprivation measures relative levels of socioeconomic deprivation in small areas of England called *lower super output areas* 20 and was linked via patient postcode by a trusted third party in CPRD.

We also conducted 2 sensitivity analyses. First, because stroke events identified only as being from CPRD and without proximal hospital admission within 30 days afterward may be existing diagnoses and not be incident cases, we conducted a sensitivity analysis excluding women with such events. Second, because patients with ICH or SAH may start their antithrombotic treatment within 60 days of diagnosis, we used a more strict definition for IS and conducted a second sensitivity analysis excluding women with unspecified stroke and prescribed an antiplatelet or anticoagulant after 7 days of diagnosis as having IS.

In addition, for women with first stroke diagnosed in time associated with pregnancy, we also examined their medical records for diagnoses of any hypertensive disorders in pregnancy, including preexisting hypertension before pregnancy, gestational hypertension, preeclampsia, and eclampsia. In addition, for women with first stroke diagnosed around the time of delivery, we examined whether or not they had preterm birth (before 37 completed weeks of gestation) because this could give us some indication about the timing of stroke related to delivery. According to the CPRD license agreement, we did not report data for cells with <5 patients. Missing data for socioeconomic deprivation were included in separate categories. Data analysis was performed using Stata/MP 11.2 (StataCorp). The study protocol was approved by the independent scientific advisory committee for database research (ISAC protocol no. 15_083R). No other ethics approval was needed for this study.

## Results

### Participant Characteristics

We identified 2 046 048 women of childbearing age with no evidence of stroke prior to our study period (Figure). Table 1 shows the basic characteristics of the study population. The median length of follow‐up was 3.4 years (interquartile range 1.4–7.5 years). Among these women, 337 297 had ≥1 live birth or stillbirth during the study period. The median age at delivery was 30.1 years (interquartile range 25.5–34.1 years). A total of 2511 women had a first incidence of stroke during the study period with 1625 (64.7%) first recorded in HES. Of the 886 first identified from CPRD, 133 (15.0%) had hospital admission records from HES within 30 days (the median time difference was 3 days, interquartile range 1–12). Of the total women with stroke, 1109 (44.2%) had IS, 705 (28.1%) had SAH, and 368 (14.7%) had ICH. There were 329 women (13.1%) with unspecified type of stroke, including 13 (0.5%) with multiple types where the primary diagnosis could not be identified.

**Figure 1:**
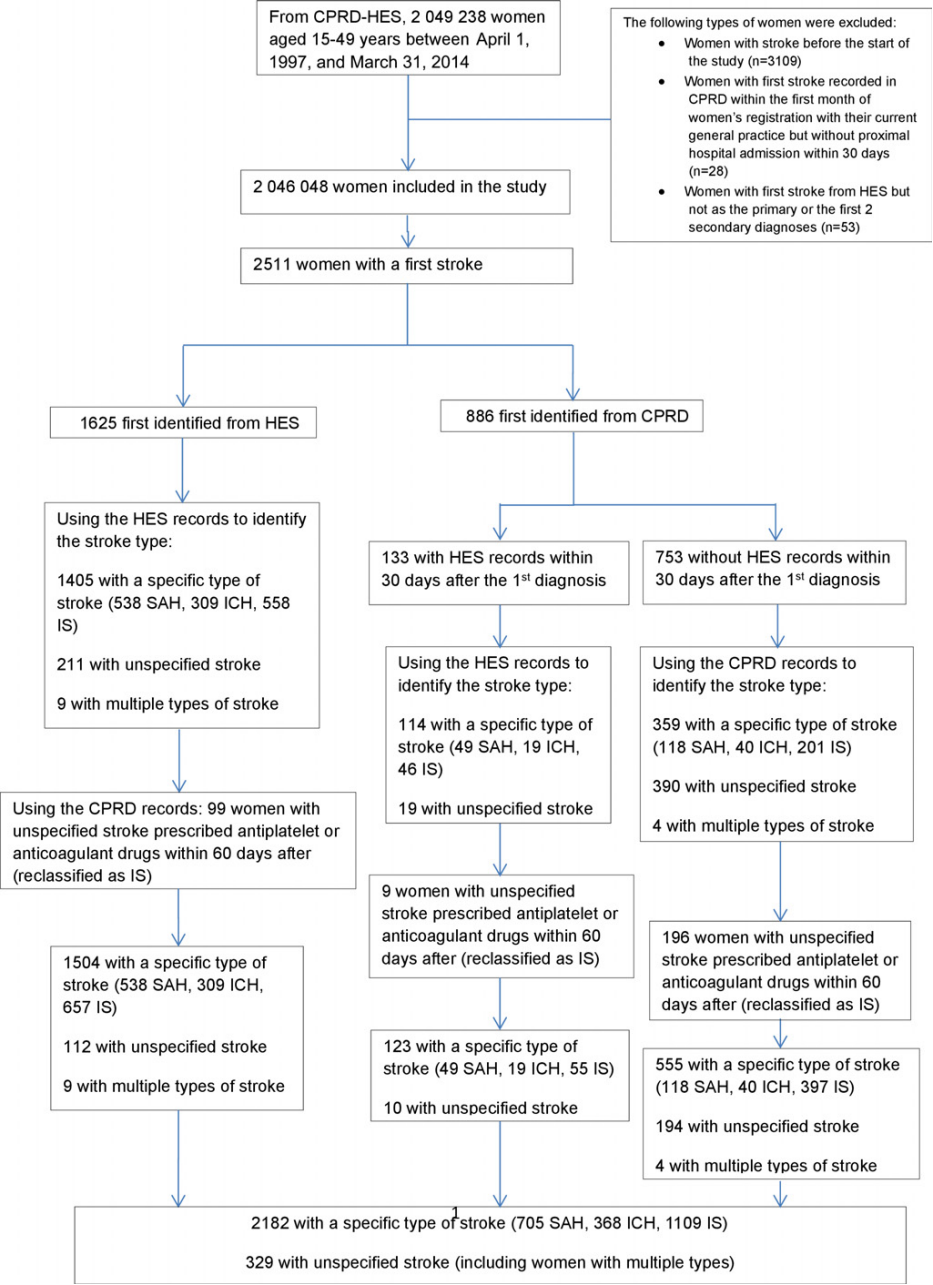
Defining study population and stroke types using both Hospital Episode Statistics (HES) and Clinical Practice Research Datalink (CPRD). ICH indicates intracerebral hemorrhage; IS, ischemic stroke; SAH, subarachnoid hemorrhage.

**Table 1:** Basic Characteristics of Study Population

Variables	n (%)
Included women in the study	2 046 048
Median follow‐up (interquartile range) in years	3.4 (1.4–7.5)
Pregnancy outcome (total pregnancies n=453 800)	
Live births	451 765 (99.55)
Stillbirths	2035 (0.45)
Study follow‐up time in person‐years (total person‐years=10 170 974)	
Nonpregnant time	9 759 438 (95.95)
Antepartum	308 378 (3.03)
Peripartum	4967 (0.05)
Postpartum	98 191 (0.97)
Cases of first stroke (total cases=2511)	
Nonpregnant time	2440 (97.17)
Pregnant time	71 (2.83)

### Absolute and Relative Risk of Stroke

The rate of first stroke in women of childbearing age was 24.7 per 100 000 person‐years (95% CI 23.7–25.7). Table 2 shows the absolute rates of stroke per 100 000 person‐years in antepartum, peripartum, postpartum, and nonpregnant time. The incidence rate was 10.7 (95% CI 7.6–15.1) per 100 000 person‐years in antepartum, similar across trimesters, and 14.2 (95% CI 6.8–29.7) in late postpartum but was higher around the time of delivery (161.1, 95% CI 80.6–322.1) and in early postpartum (47.1, 95% CI 31.3–70.9). Compared with nonpregnant time (25.0, 95% CI 24.0–26.0), women were 9 times more likely to develop first stroke in peripartum (IRR 9.4, 95% CI 4.7–18.8) and 3 times more likely in early postpartum (IRR 2.7, 95% CI 1.8–4.1) after adjustment for maternal age, socioeconomic deprivation, and calendar time (Table 2). We also found a slightly lower incidence rate of stroke in antepartum compared with nonpregnant time (IRR 0.6, 95% CI 0.5–0.9).

**Table 2:** Incidence Rates (Per 100 000 Person‐Years) of Stroke in Antepartum, Peripartum, Postpartum, and Nonpregnant Time (n=2 046 048)

All Strokes	Stroke Cases, n	Person‐Years	Rate (Per 100 000 Person‐Years)	95% CI	Adjusted Rate Ratio^a^	95% CI
Nonpregnant time^b^	2440	9 759 438	25.0	24.0–26.0	Reference	
Antepartum	33	308 378	10.7	7.6–15.1	0.6	0.5–0.9
First trimester	8	94 973	8.4	4.2–16.8	0.5	0.3–1.0
Second trimester	13	119 521	10.9	6.3–18.7	0.7	0.4–1.1
Third trimester	12	93 884	12.8	7.3–22.0	0.8	0.4–1.3
Peripartum	8	4967	161.1	80.6–322.1	9.4	4.7–18.8
Postpartum	30	98 191	30.6	21.4–43.7	1.8	1.2–2.5
Early postpartum	23	48 796	47.1	31.3–70.9	2.7	1.8–4.1
Late postpartum	7	49 395	14.2	6.8–29.7	0.8	0.4–1.7

a Rate ratio adjusted for maternal age, socioeconomic deprivation, and calendar time.

b Excluding the first 12 weeks postpartum.

### Risk of Stroke in Pregnancy and by Stroke Type

There were 71 women with first stroke in the time associated with pregnancy, 17 of whom had diagnoses of hypertensive disorders in pregnancy including 7 with diagnoses of preeclampsia or eclampsia. Eight women had stroke around the time of delivery, of which half had diagnoses of hypertensive disorders in pregnancy (37.5% with preeclampsia or eclampsia), and 12.5% had preterm delivery. In time associated with pregnancy, the most common type of stroke was IS (50.7%) followed by SAH (19.7%) and ICH (15.5%), and a similar pattern was observed in nonpregnant time. Table 3 shows the incidence rates of different types of stroke. Compared with nonpregnant time, the incidence of IS was higher in peripartum (IRR 8.0, 95% CI 2.6–25.0) and in early postpartum (IRR 4.1, 95% CI 2.4–6.8) but not in antepartum (IRR 0.6, 95% CI 0.3–1.0) or late postpartum (IRR 1.3, 95% CI 0.5–3.2) periods. In terms of hemorrhagic stroke, SAH had increased incidence in peripartum (IRR 12.9, 95% CI 4.1–40.1) but not in other time periods, whereas ICH showed increased incidence only in early postpartum (IRR 3.6, 95% CI 1.5–8.7).

**Table 3:** Incidence Rates of Different Types of Stroke in Antepartum, Peripartum, Postpartum, and Nonpregnant Time (n=2 046 048)

	Ischemic Stroke		Intracerebral Hemorrhage		Subarachnoid Hemorrhage		Unspecified Stroke	
	Cases, n	Rate^a^ (95% CI)	Cases, n	Rate^a^ (95% CI)	Cases, n	Rate^a^ (95% CI)	Cases, n	Rate^a^ (95% CI)
Nonpregnant time^b^	1073	11.0 (10.4–11.7)	357	3.7 (3.3–4.1)	691	7.1 (6.6–7.6)	319	3.3 (2.9–3.7)
Antepartum	13	4.2 (2.4–7.3)	<5	1.3 (0.5–3.5)	9	2.9 (1.5–5.6)	7	2.3 (1.1–4.8)
Peripartum	<5	60.4 (19.5–187.4)	0		<5	60.4 (19.5–187.4)	<5	40.3 (10.1–161.2)
Early postpartum	15	30.8 (18.5–51.0)	5	10.3 (4.3–24.6)	<5	4.1 (1.0–16.4)	<5	2.1 (0.3–14.6)
Late postpartum	5	10.1 (4.2–24.3)	<5	4.1 (1.0–16.2)	0		0	

According to the data license agreement, we do not report data for cells with <5 patients; therefore, we use <5 instead.

a Rate per 100 000 person‐years.

b Excluding the first 12 weeks postpartum.

### Risk of Stroke by Different Covariates

Table 4 shows the relative risk of stroke in peripartum and early postpartum compared with nonpregnant time by maternal age, socioeconomic deprivation, and calendar time. The incidence rate of stroke increased with age and socioeconomic deprivation. The incidence rate was 4.8 per 100 000 person‐years (95% CI 4.0–5.8) at ages 15 to 24 and 41.0 (95% CI 39.2–42.8) at ages 35 to 49 years in nonpregnant time, and increased risks were also observed in peripartum and early postpartum (Table 4). The relative risk of stroke in peripartum and early postpartum compared with nonpregnant time, however, was much higher in women aged 15 to 24 years (IRR 11.9, 95% CI 5.5–25.6) than in women aged 35 to 49 years (IRR 2.1, 95% CI 1.1–4.0) (Table 4). For socioeconomic deprivation and calendar time, the relative risk of stroke in peripartum and early postpartum compared with nonpregnant time was broadly similar across different groups (Table 4).

**Table 4:** Incidence Rates of Stroke in Nonpregnant Time and Combination of Peripartum and Early Postpartum by Maternal Age, Socioeconomic Status, and Calendar Time

	Nonpregnant Time^a^		Peripartum and Early Postpartum		Adjusted Rate Ratio^b^ (95% CI)
	Stroke Cases, n	Rate^c^ (95% CI)	Stroke Cases, n	Rate^c^ (95% CI)	
Maternal age, y					
15–24	110	4.8 (4.0–5.8)	7	57.9 (27.6–121.4)	11.9 (5.5–25.6)
25–34	379	13.9 (12.6–15.4)	15	48.8 (29.4–80.9)	3.6 (2.1–6.0)
35–49	1951	41.0 (39.2–42.8)	9	82.4 (42.9–158.3)	2.1 (1.1–4.0)
Index of multiple deprivation^d^					
Quintile 1 (least deprived)	405	18.0 (16.4–19.9)	<5	26.2 (8.5–81.3)	1.9 (0.6–5.8)
Quintile 2	467	21.7 (19.8–23.7)	6	55.2 (24.8–122.8)	3.5 (1.6–7.8)
Quintile 3	444	23.1 (21.0–25.4)	9	89.2 (46.4–171.5)	5.4 (2.8–10.4)
Quintile 4	554	29.3 (27.0–31.9)	5	46.2 (19.2–110.9)	2.5 (1.0–6.0)
Quintile 5 (most deprived)	562	36.7 (33.8–39.9)	8	76.6 (38.3–153.2)	3.9 (1.9–7.9)
Calendar time					
1997–2002	650	25.8 (23.8–27.8)	6	49.8 (22.4–110.9)	3.0 (1.3–6.7)
2003–2008	949	24.5 (23.0–26.2)	13	60.2 (34.9–103.6)	3.6 (2.1–6.2)
2009–2014	841	25.0 (23.3–26.7)	12	59.7 (33.9–105.1)	3.4 (1.9–6.0)

According to the data license agreement, we do not report data for cells with <5 patients; therefore, we use <5 instead.

a Excluding the first 12 weeks postpartum.

b Rate ratio mutually adjusted for other covariates (maternal age, socioeconomic deprivation, and calendar time).

c Rate per 100 000 person‐years.

d 4798 women with missing information.

### Sensitivity Analyses

In the first sensitivity analysis, we excluded 753 women with stroke identified from CPRD without hospital admission information for stroke from HES within 30 days. Similar to results from the main analysis, we found increased incidence of stroke around the time of delivery (IRR 11.8, 95% CI 5.6–24.7) and early postpartum (IRR 3.4, 95% CI 2.2–5.3) but slightly lower incidence of stroke in the antepartum (IRR 0.6, 95% CI 0.4–0.9) compared with nonpregnant time periods (Table 5). In the second sensitivity analysis, we excluded 229 women from the ischemic stroke group if they had unspecified stroke and were prescribed an antiplatelet or an anticoagulant after 7 days of diagnosis. The increased relative risk of stroke around the time of delivery and early postpartum was broadly similar to the main analysis (Table 6).

**Table 5:** Sensitivity Analyses After Excluding 753 Women With Stroke Recorded in Their General Practice Record But No Hospital Admission Within 30 Days: Incidence Rates of Stroke in Antepartum, Peripartum, Postpartum and Nonpregnant Periods

All Strokes	Stroke Cases, n	Person‐Years	Rate (Per 100 000 Person‐Years)	95% CI	Adjusted Rate Ratio^a^	95% CI
Nonpregnant time^b^	1704	9 755 854	17.5	16.7–18.3	Reference	
Antepartum	22	308 300	7.1	4.7–10.8	0.6	0.4–0.9
First trimester	7	94 948	7.4	3.5–15.5	0.6	0.3–1.3
Second trimester	7	119 491	5.9	2.8–12.3	0.5	0.2–1.0
Third trimester	8	93 861	8.5	4.3–17.0	0.7	0.4–1.4
Peripartum	7	4965	141.0	67.2–295.7	11.8	5.6–24.7
Postpartum	25	98 165	25.5	17.2–37.7	2.1	1.4–3.1
Early postpartum	20	48 783	41.0	26.5–63.5	3.4	2.2–5.3
Late postpartum	5	49 382	10.1	4.2–24.3	0.8	0.3–2.0

a Rate ratio adjusted for maternal age, socioeconomic deprivation and calendar time.

b Excluding the first 12 weeks postpartum.

**Table 6:** Sensitivity Analysis Excluding 229 Women With Unspecified Stroke But With a Prescription of an Antiplatelet or Anticoagulant After 7 Days of Diagnosis From the Ischemic Stroke Group: Incidence Rates of Ischemic Stroke in Antepartum, Peripartum, Postpartum, and Nonpregnant Time

	Ischemic Stroke		Adjusted Rate Ratio^b^ (95% CI)
	Cases, n	Rate^a^ (95% CI)	
Nonpregnant time^b^	847	8.7 (8.1–9.3)	1
Antepartum	11	3.6 (2.0–6.4)	0.6 (0.3–1.1)
Peripartum	<5	40.3 (10.1–161.1)	6.4 (1.6–25.6)
Early postpartum	15	30.8 (18.5–51.0)	4.8 (2.9–8.1)
Late postpartum	5	10.1 (4.2–24.3)	1.6 (0.7–3.8)

According to the data license agreement, we do not report data for cells with <5 patients; therefore, we use <5 instead.

a Rate per 100 000 person‐years.

b Rate ratio adjusted for maternal age, socioeconomic deprivation and calendar time.

Excluding the first 12 weeks postpartum.

## Discussion

### Principal Findings

Using data from a large population‐based cohort, we found that compared with nonpregnant time, the risk of first stroke was slightly lower in antepartum but 9‐fold higher in peripartum and 3‐fold higher in the first 6 weeks postpartum. The risks of ischemic and hemorrhagic stroke were both increased during the high‐risk period. In addition, the risk of first stroke was increased by age and socioeconomic deprivation. The relative increased risk of stroke in peripartum and early postpartum periods was much higher in women at younger age than at older age.

### Strengths and Weaknesses in Relation to Other Studies

Our study used information on >2 million women aged 15 to 49 years from England, providing the most comprehensive and contemporary estimates for stroke risks in antepartum (by trimester), early and late postpartum, and time outside these pregnancy‐related periods in women of childbearing age. Most previous research estimating the risk of stroke in women of childbearing age has been focused on either pregnancy or periods of different lengths after delivery or has not explored the risk relative to nonpregnant women of similar age. The UK Obstetric Surveillance System (UKOSS) conducted a study between 2007 and 2010^3^ and found that the risk of stroke was 1.5 per 100 000 deliveries (95% CI 1.0–2.1) and was higher for ischemic stroke than for hemorrhagic stroke.^3^ This estimate was substantially lower than our study as well as studies published in other countries or areas.^4^, ^5^, ^6^ The difference may be due to the fact that the UKOSS study only included women with stroke in antepartum and not the period soon after pregnancy. Our finding of increased risk of ischemic and hemorrhagic stroke in early postpartum compared with nonpregnancy time is in line with previous studies from Japan,^21^ Taiwan,^22^ and the United States,^9^, ^23^ utilizing hospital discharge records.

In addition, very few studies have been able to examine the period around the time of delivery. A recent study published by Kamel et al in 2014, for example, contained >1.6 million patients identified from US hospital discharge records and found that the risk of stroke was 119 per 100 000 deliveries in the first 6 weeks after delivery (approximately equivalent to 91.5 per 100 000 person‐years).^8^ However, this US study did not separate the risk around the time of delivery from the rest of the early postpartum period. The only previous study that specifically examined the stroke risk around the time of delivery was a Swedish study^6^ published in 2001. This study used the Swedish Birth Register linked with the Inpatient Register between 1987 and 1995 and found that, compared with the risk in the baseline period (which included the period before pregnancy and the first 2 trimesters), the risk of stroke was higher around delivery and in the first 6 weeks postpartum.^6^ Importantly, this Swedish study included deliveries only between 1987 and 1995 and did not report the risk in the first or second trimester. Our study results are broadly in line with the Swedish study but provide more contemporary and comprehensive estimates for the stroke risk (including in first and second trimesters).

Regarding specific type of stroke, in line with our results, a much older US study published in 1996 using hospital discharge records found that the risks of IS and ICH were both increased in the first 6 weeks after delivery compared with nonpregnant time.^23^ Another more recent US study solely focusing on ICH found that the risk of ICH was 7.1 per 100 000 person‐years compared with 5.0 in the general population of similar age but did not provide the estimate for other types of stroke.^24^ Swedish studies found that the risks of IS, ICH, and SAH were all increased around the time of delivery compared with the period before pregnancy and the first 2 trimesters.^6^, ^7^ Nevertheless, we did not find any women with ICH around the time of delivery probably due to lack of study power for specific types of stroke, and particularly for ICH.

A limitation of this study is potential misclassification of the incident stroke. First, the median length of prospective follow‐up was only 3.4 years (interquartile range 1.4–7.5); therefore, we might have misclassified a recurrent stroke as the first stroke; however, we investigated historical stroke events (wherever available) and excluded women with stroke before start of the study in the main analysis. Second, 753 women were diagnosed with stroke in primary care without admission to hospital within 30 days. Because such diagnoses might not indicate “incident” stroke events, we performed a sensitivity analysis by excluding these women. The absolute rates of stroke for each period during and outside pregnancy reduced slightly but remained largely within the confidence limits of the original analysis. The relative rates during pregnancy‐related time compared with nonpregnant time were extremely similar to the main analysis, so this did not change the interpretation. Third, because we have used only HES to identify pregnancies, pregnancies ending earlier (not ending in live birth or stillbirth) were not included in this study. Consequently, if a woman had a stroke event in pregnancy that did not end in a delivery, such stroke event would be included in nonpregnant time. Fourth, we should acknowledge that we used date of hospital admission as the date of stroke diagnosis, which means there might be some misclassification between antepartum and postpartum stroke; however, by creating a third category of *peripartum*, around the time of delivery, we believe we reduced its impact.

In addition, we relied on clinician diagnosis to identify stroke, and there was no information on computed tomography brain scan or other etiological information to validate the stroke diagnosis or allow a mechanism‐based classification of stroke types. Hospitals in England will use computed tomography and other tests to classify the stroke diagnosis for management and coding in HES, but we were not able to access this information directly, and it would not be standardized across all of our cases. For a small proportion of women without stroke type specified at the time of their first stroke diagnosis, prescriptions of antiplatelet and anticoagulant drugs within 60 days after stroke diagnosis were used to classify IS. By using this approach, it is possible that we might have misclassified some women as having ischemic stroke when they did not; however, we believe this is unlikely for a number of reasons. First, the majority of stroke cases during the time around delivery and early postpartum were identified from HES based on ICD‐10 codes with stroke type specified. Second, the use of an antiplatelet or anticoagulant after a stroke for other than IS would be very unlikely; however, we also conducted a sensitivity analysis that excluded women with unspecified stroke who were prescribed an antiplatelet or anticoagulant after 7 days of diagnosis from the ischemic stroke group. We found broadly similar results to the main analysis.

### Possible Explanations and Implications for Clinicians and Researchers

Our study found that the risk of both ischemic and hemorrhagic stroke was increased around the time of delivery and in the first 6 weeks postpartum. In addition, our results suggested that the mechanisms of increased relative risk around the time of delivery and early postpartum are age‐dependent, and younger women had a higher relative risk of stroke in the high‐risk period than older women. Certain delivery‐related factors (eg, the prothrombotic state) could have substantial impact on the occurrence of ischemic stroke in the high‐risk period. Previous research has found that the risk of first venous thromboembolism is also significantly higher around the time of delivery and early postpartum.^17^ In addition, other risk factors unique to pregnancy, such as pregnancy‐induced hypertension and preeclampsia or eclampsia, especially increase the risk of hemorrhagic stroke.^25^, ^26^, ^27^, ^28^ In our study, ≈25% of women with stroke in the time associated with pregnancy had diagnoses of hypertensive disorders in pregnancy, much higher than risk expected in the general population, indicating that hypertensive disorders in pregnancy is an important risk factor for pregnancy‐related stroke. In contrast, blood pressure normally falls during the first half of pregnancy, which could reduce the risk of hemorrhagic stroke in early pregnancy. In addition, the universal use of free, routinely provided antenatal care in the United Kingdom starting from early in the first trimester could help identify young women with traditional risk factors for stroke and might lead to monitoring and treatment of high blood pressure in pregnancy. Outside of pregnancy, such women of the same age will less often come into contact with health professionals. Healthcare professionals should be aware of the pregnancy‐related stroke risk and provide women with immediate investigation and treatment if stroke symptoms develop. Prospective registries recording stroke events in and after pregnancy, particularly relative to delivery and labor, could be potentially beneficial in monitoring and understanding occurrence of stroke in pregnant women.

## Sources of Funding

Bath is Stroke Association Professor of Stroke Medicine, and is a National Institute of Health Research Senior Investigator. Stephansson was supported by the Swedish Research Council (grant 2013‐2429).

## Disclosures

Nelson‐Piercy has received consultancy fees from Alliance Pharmaceuticals, UCB and lecturing fees from UCB, Sanofi and Leo‐Pharma. No other conflicting interests have been declared.
